# Energy-Consistent Neural Networks with Fenchel–Young Loss for Physics-Guided Energy Prediction in Sheet Metal Forming Under Small-Data Conditions

**DOI:** 10.3390/ma19081571

**Published:** 2026-04-14

**Authors:** Seong-Su Jhang, Jae-Young Kwon, Won-Hee Lee, Hong-Gyu Park

**Affiliations:** 1 Department of ICT Convergence Research Center, Korea Electronics Technology Institute, Changwon-si 51394, Republic of Korea; jss0221@keti.re.kr (S.-S.J.);; 2 Department of Autonomous Manufacturing Research Center, Korea Electronics Technology Institute, Seongnam-si 13509, Republic of Korea; 3 Department of Electronic Engineering, Changwon National University, Changwon-si 51140, Republic of Korea

**Keywords:** sheet metal forming, energy-informed loss, physics-constrained learning, neural networks, Fenchel–Young loss function

## Abstract

This study addresses energy-response prediction in sheet metal forming under small-data conditions, where conventional simulation-based approaches are computationally expensive and data acquisition is limited. We propose an Energy-Informed Neural Network (EINN) framework that integrates energy consistency constraints and a Fenchel–Young duality-based loss to enforce physically consistent learning without relying on explicit governing equations. Using a dataset generated from 54 finite element simulations across 18 materials and three friction conditions, the proposed model demonstrates significant performance improvements. Specifically, EINN achieves an RMSE of 0.0096, MAE of 0.0065, and R^2^ of 0.9778, corresponding to approximately a 48% reduction in RMSE compared to the best baseline model. Compared to an energy-constrained neural network without the Fenchel–Young term, prediction error is reduced by approximately 50% with substantially improved stability. These results indicate that embedding energy-consistent dual structures enhances both prediction accuracy and robustness, providing a practical surrogate modeling approach for process optimization in sheet metal forming under limited data availability.

## 1. Introduction

### 1.1. Finite Element-Based Approaches in Sheet Metal Forming

Sheet metal press forming processes, such as deep drawing and bending, are representative mass-production technologies widely employed in the automotive, home appliance, and aerospace industries. With increasing demands for lightweight structures, the application of high-strength materials, and growing geometric complexity of products, the importance of accurate process design and quality prediction in sheet metal forming has become more pronounced. However, sheet metal forming inherently involves highly nonlinear process behavior due to the simultaneous coupling of contact conditions, large deformations, and nonlinear material plasticity [1,2]. In particular, variations in lubrication conditions and material properties have a direct impact on forming loads, deformation distributions, energy dissipation, and fracture occurrence, thereby increasing uncertainty during the process design stage [1,3,4,5,6,7,8,9,10,11,12,13,14,15].

In industrial practice, trial-and-error-based process design is still frequently adopted, which often leads to substantial costs associated with process redesign, scrap losses, and prolonged development cycles [1]. To address these challenges, finite element method (FEM)-based forming simulations have been widely used for process design and optimization [4,10,16]. FEM is widely regarded as a reliable and physically grounded tool for predicting deformation behavior, stress–strain distributions, and energy responses during forming processes. Nevertheless, in practical applications, the effectiveness of FEM simulations may depend on the formulation of constitutive models and the accuracy of material parameter calibration [1,3,9,17,18,19]. In real forming operations, multiaxial loading conditions and variations in deformation paths are common, which can make it challenging to fully capture material behavior using standard material tests such as uniaxial tension or simple shear tests [1,3,6,8,9,10]. Furthermore, when a wide range of material properties, friction conditions, and lubrication states must be considered, repeated FEM analyses can require considerable computational time and resources, which may limit their efficiency in extensive process parameter exploration and optimization [16,20]. Also, emerging advanced metallic materials such as high-entropy alloys (HEAs) have attracted increasing attention in metal forming and processing fields due to their exceptional mechanical properties and microstructural tunability. In particular, rolling- and surface deformation-based processes have been actively investigated to tailor gradient microstructures and enhance strength–ductility synergy in HEAs [21]. These developments highlight the growing importance of advanced material systems in modern forming applications. Accordingly, increasing material complexity and process variability have led to a growing need for comprehensive materials engineering analysis that can capture the coupled effects of material properties, deformation paths, and contact conditions. In sheet metal forming processes such as press forming and rolling, accurate prediction of deformation behavior requires not only conventional empirical approaches but also physics-based modeling and energy-consistent analysis frameworks. While finite element analysis (FEA) has been widely adopted as a reliable tool for process simulation, its effectiveness strongly depends on accurate material modeling and parameter calibration, particularly under multiaxial and path-dependent deformation conditions. In addition, traditional formability evaluation methods such as forming limit diagrams (FLDs) suffer from inherent limitations due to their strain-path dependency, which restricts their applicability to complex forming scenarios. These challenges highlight the need for advanced modeling approaches that can overcome the limitations of conventional FLD-based criteria while complementing FEA in capturing complex forming behavior.

### 1.2. Formability Evaluation Using FLD and Its Limitations

From a formability evaluation perspective, the forming limit diagram (FLD) has been widely adopted as an industrial standard for assessing material formability in sheet metal forming processes. FLD provides a convenient representation of forming limits under different strain paths and has been extensively used in both industrial practice and academic research. However, FLD is inherently path-dependent, which can limit its applicability to complex forming processes involving non-proportional loading conditions. This characteristic makes reliable formability prediction challenging in practical scenarios where deformation histories vary significantly. To address these limitations, alternative criteria such as forming limit stress diagrams (FLSD/FLSC) and damage- or energy-based indicators have been proposed to complement conventional FLD-based approaches [2,8,9,10].

These approaches are therefore considered complementary to FEM, enabling more flexible and efficient evaluation of forming conditions rather than replacing conventional simulation-based methods.

### 1.3. Data-Driven and Physics-Informed Learning Approaches

Against this background, recent research has actively explored the integration of simulation and experimental data with machine learning (ML) techniques to address tasks such as material property identification, process optimization, and quality prediction. In particular, neural network-based approaches have demonstrated promising applicability in the field of sheet metal forming [1]. Recent studies have also demonstrated that deep learning models can be effectively applied even under small-data conditions when appropriate strategies such as data augmentation or generative modeling are employed. For example, a VAE-based approach [22] has been used to generate synthetic data, significantly improving prediction performance in hydrogen yield estimation under limited datasets. These findings highlight the potential of combining generative models with predictive learning to overcome data scarcity challenges.

More recently, physics-informed neural networks (PINNs) have been introduced to incorporate physical constraints into the learning process, improving generalization performance under limited data conditions [5,20,23]. By embedding governing equations into the loss function, PINNs enhance physical consistency compared to purely data-driven models. However, several challenges remain. As the governing physics becomes more complex, computational costs increase significantly due to repeated evaluations of high-order derivatives. Moreover, strong-form PDE-based approaches face difficulties in handling contact conditions and path-dependent plasticity, which involve non-smooth and history-dependent behavior.

To address these limitations, expert-informed neural networks (EINNs) have been proposed, where domain knowledge is incorporated into the training objective through additional constraint terms [16]. While such approaches improve predictive robustness, many existing formulations rely on heuristic constraints and lack a clear theoretical foundation in terms of energy consistency or convex structure. In this study, we propose an energy-informed learning framework based on the Fenchel–Young formulation, which introduces a convex duality structure into the loss function. By embedding energy consistency as a structural constraint, the proposed approach enforces compatibility between physically conjugate variables such as velocity, momentum, and kinetic energy. This formulation enhances both learning stability and physical interpretability, particularly in small-data environments where overfitting and physically inconsistent predictions may arise. Table 1 summarizes the conceptual differences between classical PINNs, general energy-based learning frameworks, previously proposed EINN approaches, and the proposed formulation. Unlike conventional PINNs that rely on explicit PDE residuals, the proposed method enforces physical consistency through a convex-duality-based energy structure without requiring explicit governing equations. This distinction enables stable learning in complex forming problems involving contact and path-dependent plasticity.

## 2. Methodology

### 2.1. Sheet Metal Forming FEA

In this study, finite element analysis (FEA) was used to quantitatively analyze the energy behavior occurring in sheet metal forming processes and to utilize the results for model training. The dome forming-based simulation was adopted as the representative forming process in this study, as it enables the reproduction of complex deformation states observed in practical sheet metal forming. In general, forming limit diagrams (FLDs) are determined using standardized experimental methods such as the Marciniak test (in-plane deformation) and the Nakazima test (out-of-plane dome test) [10]. The Nakazima test employs a hemispherical punch under lubricated conditions, allowing the realization of various strain paths by adjusting specimen geometry and friction conditions, although it remains dependent on strain paths. In particular, dome forming induces biaxial tensile deformation at the pole region while simultaneously involving material inflow and frictional effects at the flange region, thereby enabling comprehensive observation of deformation and failure behavior. Previous studies have shown that dome forming-based FE models can effectively reproduce experimental deformation histories and failure mechanisms [25]. The hemispherical punch generates a biaxial tensile stress state at the center of the sheet, which is a representative condition where necking and fracture occur in practical forming processes. Moreover, dome forming is not limited to a localized fracture test; it allows simultaneous observation of multiple phenomena, including central tensile-dominated deformation, edge drawing behavior, frictional interactions, and material flow variations influenced by blank holder force [25].

**Figure 1:**
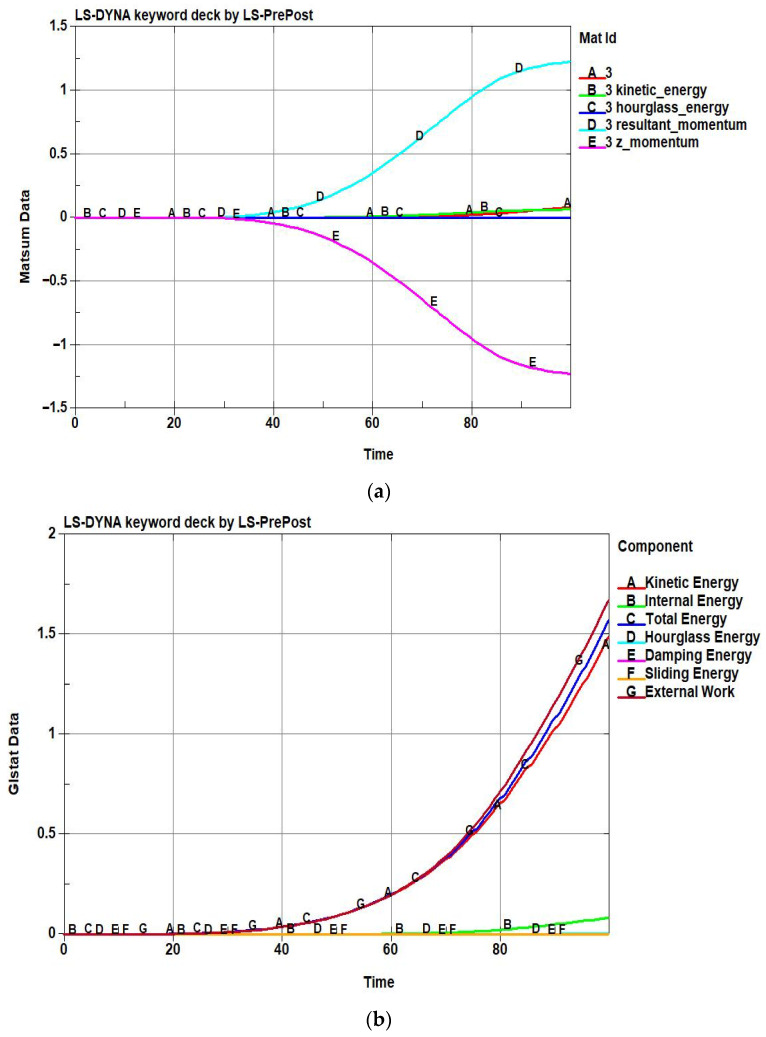
Energy responses obtained from the dome forming simulation: (**a**) Material-level energy and momentum histories of the blank extracted from the matsum output. The curves represent internal energy (A), kinetic energy (B), hourglass energy (C), resultant momentum (D), and z-direction momentum (E). The internal energy dominates the response as plastic deformation accumulates, while kinetic and hourglass energies remain comparatively small, indicating a quasi-static and numerically stable forming process.; (**b**) Global energy balance of the forming system obtained from the glstat output, including kinetic energy (A), internal energy (B), total energy (C), hourglass energy (D), damping energy (E), sliding (contact) energy (F), and external work (G). The close agreement between the total energy and the external work confirms physical energy consistency throughout the forming process, while bounded hourglass and damping energies demonstrate numerical stability.

The finite element simulations were performed using LS-DYNA with an explicit dynamic formulation under quasi-static conditions. The blank was modeled using shell elements with an appropriate elastoplastic material model, while contact interactions between the blank and forming tools were defined using a penalty-based contact algorithm. Boundary conditions, including punch motion, blank holder force, and friction coefficients, were prescribed to represent dome forming conditions. Energy-related outputs were extracted from standard LS-DYNA databases (MATSUM and GLSTAT) for subsequent analysis. Through the simulations, the time-dependent responses of internal energy, kinetic energy, and total energy under varying forming conditions were systematically collected and used as learning data. Figure 1a,b presents the time histories of energy responses extracted from the LS-DYNA simulations for the blank material. Figure 1a shows the time histories of internal energy, kinetic energy, hourglass energy, and momentum components of the blank material. Figure 1b presents the system-level energy components, including kinetic energy, internal energy, sliding energy, external work, damping energy, and total energy. These results confirm that the energy data of the blank material used for training the Energy-Informed Neural Network were generated under physically consistent and numerically stable conditions at both the material and system levels.

The material properties of each sheet metal were defined based on representative values of metallic materials widely used in actual industrial forming applications. As summarized in Table 2, the materials considered in the finite element analysis include a total of 18 metallic materials: commercially pure aluminum (Al 1100), stainless steel (STS304), galvanized steel sheet (GI), cold-rolled steel sheet (SPCC), hot-rolled steel sheet (SS400), magnesium alloy (AZ31B), copper (Cu), brass, tin-plated steel sheet (SPTE), titanium (Ti Grade 2), dual-phase high-strength steel (DP590), beryllium copper (BeCu), Inconel 718, mild steel (SS41 equivalent), aluminum alloys (AA5052-O, AA6061-T4, and AA7075-T6), and DC01 steel sheet.

Three friction conditions representing typical lubrication states in sheet metal forming were defined and employed as control parameters to capture the differences in energy behavior associated with lubrication conditions rather than to reproduce material-specific tribological properties. The corresponding coefficients of friction (CoF) were determined based on representative values reported in the literature. The considered conditions include an unlubricated (dry) condition, a generally lubricated condition, and a well-lubricated condition, with CoF values of 0.4, 0.2, and 0.05, respectively. These three friction levels were selected as representative values within experimentally reported ranges for various aluminum alloy sheets, with the primary objective of validating the physical consistency of the proposed energy-based learning framework rather than explicitly modeling material-dependent tribological variations. First, under the dry or unlubricated condition, direct contact occurs between the aluminum alloy sheet and the tool steel surface, where adhesive wear and galling dominate the friction mechanism. According to ring compression test studies evaluating shear friction coefficients, CoF values in the range of approximately 0.34–0.40 have been reported for Al 1100-O and Al 6061-T6 under dry conditions [11]. In addition, strip draw test-based investigations have shown that, depending on contact pressure and sliding speed, CoF values of approximately 0.22–0.28 are observed under dry conditions, which are significantly higher than those measured under lubricated conditions [14]. Based on these findings, a CoF of 0.4 was adopted in this study to represent the unlubricated condition. Next, the generally lubricated condition refers to forming processes in which low-viscosity mineral oils, synthetic oils, or conventional press lubricants are applied. Under these conditions, a partial lubricant film is formed, which mitigates friction and galling; however, the friction coefficient may increase again under high contact pressures or lubricant film breakdown. In lubricant evaluation studies for Al 5182-O sheets using the cup drawing test, CoF values in the range of approximately 0.08–0.12 were reported for various commercial lubricants [13]. Similarly, strip draw test-based studies reported CoF values of approximately 0.13–0.17 when conventional lubricants were used [14]. Considering these results, a conservative representative value of 0.2 was selected to characterize the generally lubricated condition in the present study.

Finally, the well-lubricated condition corresponds to forming scenarios in which high-performance lubricants, solid lubricant coatings, or dry film lubricants (DFLs) are applied. Ring compression test results indicate that the use of graphite- or PTFE-based lubricants can reduce the CoF to approximately 0.03–0.07 for Al 1100-O sheets [11]. In addition, deep drawing and stretching experiments employing boric acid-based dry film lubricants have demonstrated very low CoF values of approximately 0.04, effectively suppressing adhesive wear and galling [12]. Tribological experiments on hot-melt-based dry film lubricants have also reported consistently low friction coefficients when a stable lubricant film is maintained [15]. Based on these prior studies, a CoF value of 0.05 was selected to represent an idealized well-lubricated condition in this study.

### 2.2. Dataset and Fenchel–Young Loss-Based EINN Design

To predict the energy response and forming behavior of sheet metal forming processes using only a limited amount of simulation data, and to further utilize these predictions for process design and condition optimization, a learning framework is required that satisfies not only data fitting performance but also physical consistency. In particular, for sheet metal forming problems, it is often difficult to explicitly formulate the governing partial differential equations (PDEs), and the observable outputs are frequently restricted to global physical quantities such as energy [5,16,20,23]. As a result, the importance of physics-based loss design becomes even more pronounced. In this context, the present study adopts the Fenchel–Young loss, grounded in the theory of generalized conjugate functions, as the training loss function for an Energy-Informed Neural Network (EINN).

A total of 54 finite element simulations were conducted using LS-DYNA, constructed as a full-factorial combination of 18 distinct metal material property sets and three lubrication conditions defined by different friction coefficients. Each simulation generated one supervised sample. The input variables were designed to reflect the fundamental physical drivers of the forming process. These include intrinsic material properties such as density (ρ), Young’s modulus (E), and yield strength (σy), which govern inertia, elastic stiffness, and plastic deformation behavior. In addition, the friction coefficient was incorporated to represent interface and lubrication effects, directly influencing sliding dissipation and external work transfer. Together, these parameters define the primary mechanical and contact conditions of the forming system. To characterize variations in the system-level outputs, global physical indicators were extracted from LS-DYNA output files (GLSTAT and MATSUM). These descriptors include kinetic, internal, sliding, hourglass, and total energy components, as well as external work, rigid-body (punch) velocity and motion metrics. The predicted outputs focus on representative global energy responses of the forming process, including the final and maximum total energy, maximum kinetic energy, final internal energy, and terminal rigid-body velocity. Reproducibility and robustness under small-data conditions were addressed through a random 7:3 split of the dataset. Feature normalization was performed using statistics derived solely from the training set, preventing data leakage. Target variables were scaled consistently, and physics-based loss terms were computed after inverse transformation into physical units.

The proposed EINN is implemented as a fully connected multilayer perceptron with three hidden layers (128, 64, and 32 neurons) and ReLU activation functions. The network was optimized using the AdamW optimizer with a learning rate of 1 × 10^−3^ and a weight decay of 1 × 10^−6^. The maximum number of training epochs was set to 2000, and early stopping was employed based on validation loss.

#### 2.2.1. Global Energy Terms

In explicit finite element analysis (FEA), the global energy balance of the system at time *t* can be expressed as shown in Equation (1):

(1) $$E_{total}\left(t\right)=KE\left(t\right)+IE\left(t\right)+E_{contact}\left(t\right)+E_{hourglass}(t)$$

where $KE\left(t\right)$ denotes the kinetic energy, $IE\left(t\right)$ represents the internal energy including elastic and plastic deformation energies, $E_{contact}\left(t\right)$ corresponds to the energy dissipated or transferred through contact and friction, and $E_{hourglass}(t)$ is the numerical stabilization energy introduced to suppress zero-energy modes in reduced integration elements. This relation merely represents an accounting identity and does not guarantee kinematic–dynamic consistency among momentum, velocity, and kinetic energy. Such limitations become critical in small-data learning settings, motivating the use of a duality-based energy loss. These observations are empirically validated in the Section 3.

#### 2.2.2. Fenchel–Young Structure and Estimation of Effective Mass

In the explicit finite element simulations, the hemispherical punch was modeled as a rigid body with its motion constrained to the translational direction along the global *z*-axis. Consequently, the dominant kinetic contribution arises from this prescribed vertical motion. The one-dimensional rigid-body approximation introduced here represents a reduced-order abstraction of the punch dynamics for the purpose of defining a physically interpretable energy–momentum dual structure. Assuming the punch (rigid-body) motion can be approximated as a one-dimensional rigid-body system, the kinetic energy is defined as Equation (2):

(2) $$KE\left(v\right)=\frac{1}{2}mv^{2}$$

where v is the punch velocity and m is its effective mass. The dual variable associated with velocity is the linear momentum, as shown in Equation (3):

(3) $$p=\frac{\partial KE}{\partial v}=mv$$

Applying the Legendre–Fenchel transform to the kinetic energy yields the dual function (4)

(4) $$KE^{*}(p)=\frac{p^{2}}{2m}$$

The Fenchel–Young inequality then reads (5)

(5) $$KE\left(v\right)+KE^{*}\left(p\right)-pv\geq 0$$

The corresponding Fenchel–Young gap therefore provides a quantitative measure of kinematic–dynamic consistency.

The effective punch mass m was estimated from Equation (6) (kinetic energy–momentum relation),

(6) $$KE=\frac{p^{2}}{2m}\;\to \;m=\frac{p^{2}}{2KE}$$

The instantaneous mass m(t) was computed at each time step, and the median value over the entire time history was adopted as m. This choice mitigates the influence of numerical noise, added mass effects, and outliers associated with contact events in explicit dynamics.

#### 2.2.3. Fenchel–Young Residual and Global Energy Consistency

Using the estimated m, the Fenchel–Young residual for the punch motion was defined as Equation (7):

(7) $$\Psi_{FY}\left(t\right)=KE\left(t\right)+\frac{{p(t)}^{2}}{2m}-p\left(t\right)v\left(t\right),\;\Psi_{FY}\left(t\right)\geq 0$$

A value of $\Psi_{FY}\left(t\right)=0$ corresponds to ideal momentum–velocity–energy consistency. In the FEA simulations, the residual remained small and positive, indicating that dynamic inconsistencies induced by contact, friction, and numerical damping were limited.

In addition, global energy consistency was verified based on the system-level energy balance of the explicit finite element analysis, as expressed in Equation (8):

(8) $$∆W_{ext}\left(t\right)-∆(KE+IE+E_{contact}+E_{hourglass})$$

Which remained close to zero during stable simulation intervals. This confirms that the energy time histories used for training were generated under numerically stable and physically consistent conditions.

#### 2.2.4. Energy-Based Loss Function for Neural Network Training

The proposed Energy-Informed Neural Network (EINN) $f_{\theta }\left(\cdot \right)$ maps an input vector $x\in R^{d_{x}}$ to the output vector Equation (9):

(9) $$\hat{y}=f_{\theta }\left(x\right)=[{\hat{E}}_{total},{\hat{E}}_{max},{\hat{KE}}_{max},{\hat{IE}}_{end},{\hat{V}}_{z,end}]$$

All outputs are predicted in a normalized space for numerical stability, while physical constraints are enforced in the original physical scale.

The data fidelity loss is defined as the mean squared error Equation (10):

(10) $$L_{data}=\frac{1}{N}\sum_{i=1}^{N}{\left\|{\hat{y}}^{\left(i\right)}-y^{\left(i\right)}\right\|}_{2}^{2}$$

To ensure physical plausibility, additional energy-based constraint losses were introduced:

(11) $$\mathrm{Total}\;\mathrm{energy}\;\mathrm{consistency}:\;\left|{\hat{E}}_{total}-{\hat{E}}_{max}\right|$$

(12) $$\mathrm{Energy}\;\mathrm{hierarchy}\;\mathrm{constraint}:\;\max\left(0,{\hat{KE}}_{max}-{\hat{E}}_{max}\right);$$

(13) $$\mathrm{Non}\text{-}\mathrm{negativity}\;\mathrm{of}\;\mathrm{internal}\;\mathrm{energy}:\;max\left(0,-{\hat{IE}}_{end}\right)$$

(14) $$\mathrm{Terminal}\;\mathrm{velocity}\;\mathrm{direction}\;\mathrm{constraint}:\;max\left(0,{\hat{v}}_{z,end}\right)$$

Crucially, a Fenchel–Young-based kinetic energy constraint was incorporated to enforce the dual structure between momentum, velocity, and kinetic energy. By minimizing $\Psi_{FY}$, the network is guided to learn a physically interpretable dynamic structure rather than merely fitting numerical energy values.

The neural network predicts energy-related quantities, from which velocity and momentum are derived in the physical scale. These variables are then used to compute the Fenchel–Young residual, which directly couples the network outputs with the dual energy structure. Through this formulation, the Fenchel–Young term enforces a coupling between predicted kinetic energy, velocity, and momentum, ensuring that these variables satisfy a physically consistent dual relationship. Since the Fenchel–Young inequality is satisfied if and only if the conjugate relationship holds, minimizing the residual enforces kinematic–dynamic consistency rather than merely fitting numerical values.

The final loss function is therefore defined as Equation (15):

(15) $$L=L_{data}+\sum_{k=1}^{4}\lambda_{E_{k}}L_{E_{k}}+\lambda_{FY}\Psi_{FY}$$

where $\lambda_{E_{k}}$ and $\lambda_{FY}$ are weighting coefficients for the energy constraints and the Fenchel–Young term, respectively. The four energy-based constraint terms used in this study are defined in Equations (11)–(14). The corresponding weights $\lambda_{E_{1}}$, $\lambda_{E_{2}}$, $\lambda_{E_{3}}$, $\lambda_{E_{4}}$ were determined through a dedicated sensitivity analysis using the baseline model (MLP with energy constraints without the Fenchel–Young term). Specifically, multiple weight combinations were tested, and the configuration ($\lambda_{E_{1}}$, $\lambda_{E_{2}}$, $\lambda_{E_{3}}$, $\lambda_{E_{4}}$) = (1.2, 1.5, 1.0, 1.0) achieved the best predictive performance (RMSE = 0.0159, MAE = 0.0107, R^2^= 0.9142). This selected weight configuration was then kept fixed and applied identically to the proposed EINN, in order to ensure a fair comparison and to avoid introducing additional tuning degrees of freedom when evaluating the contribution of the Fenchel–Young term.

The weight of the Fenchel–Young loss term defined in Equation (15) was fixed to 1. This choice allows the dual-structure regularization effect to be incorporated in a balanced manner without excessively dominating the data-fitting objective. In summary, the proposed loss design aims not to merely match energy magnitudes, but to embed the underlying energy generation and transfer mechanisms into the learning process, thereby enabling physics-consistent surrogate modeling even in small-data regimes [26,27,28,29,30,31]. The Fenchel–Young residual is computed using the predicted velocity and the corresponding conjugate momentum derived from the network outputs. Specifically, the network predicts energy-related quantities, from which the velocity and momentum variables are obtained in the physical scale. The Fenchel–Young residual $\Psi_{FY}$ is then evaluated and directly incorporated into the loss function. During training, this term acts as a structural regularization, penalizing physically inconsistent combinations of velocity, momentum, and energy. Since $\Psi_{FY}$ is differentiable with respect to the network outputs, it is naturally integrated into the backpropagation process and optimized jointly with the data fidelity loss.

## 3. Results and Discussion

### 3.1. Dataset Limitation and Evaluation Metrics

The dataset represents a small-data scenario typical of high-fidelity simulation-based engineering problems, where each sample corresponds to a unique combination of material and process parameters. Although the number of samples is limited, the dataset spans a range of physically meaningful conditions, ensuring variability in the resulting energy responses. The dataset consists of input features describing material properties and process conditions, along with corresponding output variables representing key energy responses, including total energy, maximum energy values, and terminal kinematic quantities. Since the present study is based entirely on simulation-generated data, experimental measurement noise and sensor uncertainty are not explicitly incorporated. This represents an inherent limitation of the current validation framework. Nevertheless, the proposed physics-consistent formulation embeds structural energy constraints within the loss function, thereby restricting the hypothesis space to physically admissible solutions. Such structural regularization is expected to reduce sensitivity to physically inconsistent fluctuations that may arise under practical measurement uncertainty.

For comparison with conventional machine learning approaches, multiple regression models—namely multilayer perceptron (MLP), random forest (RF), k-nearest neighbors (KNN), support vector regression (SVR), and linear models—were trained. Model performance was evaluated using the mean absolute error (MAE), root mean squared error (RMSE), and coefficient of determination (R^2^), which collectively capture accuracy, robustness, and explanatory power.

Robust validation and generalization performance under unseen conditions were assessed using a repeated evaluation protocol. The dataset was divided into training and test sets with a ratio of 7:3, and model performance was further evaluated using 2-fold cross-validation repeated over five random seeds, resulting in a total of 10 independent runs. This repeated evaluation strategy provides a more reliable assessment of model robustness and generalization across different data splits. Additionally, the use of multiple random seeds reduces the risk of performance bias associated with specific data partitions.

### 3.2. Training Behavior of the Energy-Informed Neural Network

**Figure 2:**
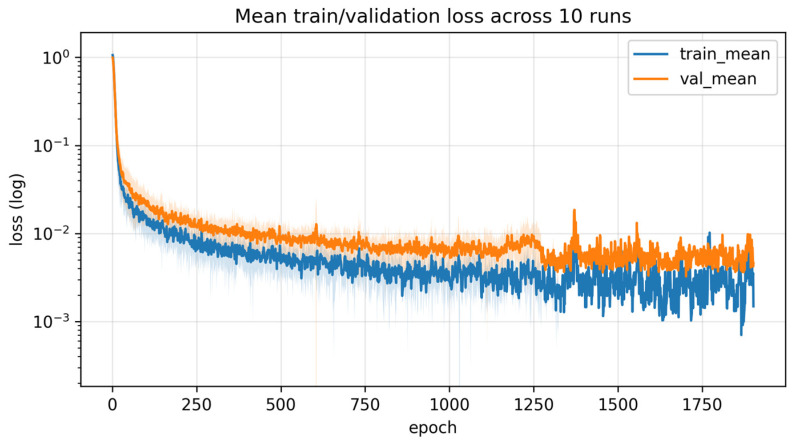
Mean training and validation loss histories of the proposed Energy-Informed Neural Network (EINN) across 10 runs, plotted on a logarithmic scale. The shaded regions indicate the standard deviation across runs.

Figure 2 presents the mean training and validation loss histories of the proposed Energy-Informed Neural Network (EINN) across 10 runs on a logarithmic scale. The total loss includes both the data fidelity term and the physics-based energy constraints, including the Fenchel–Young kinetic energy residual. As shown in Figure 2, both the training and validation losses decrease rapidly during the early stage of learning, indicating efficient fitting of the energy-response data. After this initial phase, the losses gradually decrease and stabilize, reflecting progressive refinement of the learned energy structure. The relatively smooth validation trend without noticeable divergence or fluctuation suggests that the proposed EINN does not suffer from severe overfitting despite the limited size of the training dataset. Furthermore, the shaded regions represent the standard deviation across repeated runs, demonstrating that the training process remains stable and consistent under different random initializations and data splits. The narrow variance observed in both training and validation losses confirms the robustness and reproducibility of the proposed learning framework. Overall, these results indicate that the proposed EINN achieves stable convergence while maintaining physical consistency, supporting its suitability for energy-based surrogate modeling and process condition exploration in sheet metal forming applications.

**Figure 3:**
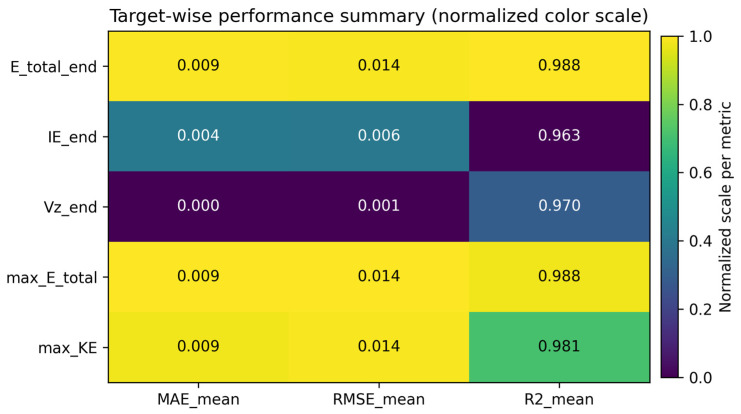
Target-wise predictive performance summary of the proposed EINN across 10 runs in terms of MAE, RMSE, and R^2^.

Figure 3 presents a heatmap summarizing the target-wise predictive performance of the proposed EINN across 10 runs in terms of MAE, RMSE, and R^2^. Each row corresponds to a target variable, while each column represents a performance metric averaged over repeated runs. The results indicate that the proposed model achieves consistently low prediction errors and high coefficients of determination across all target variables. In particular, high R^2^ values close to unity demonstrate that the model effectively captures the underlying relationships between input parameters and energy-related outputs.

Notably, the model maintains stable and accurate predictions not only for global energy quantities, such as total energy, but also for derived physical variables including internal energy, kinetic energy, and terminal velocity. This suggests that the proposed energy-consistent learning framework generalizes well across multiple physically meaningful outputs. Overall, the heatmap highlights the robustness and reliability of the proposed EINN in multi-target regression settings, further supporting its applicability as a physically interpretable surrogate model for sheet metal forming processes.

### 3.3. Performance Comparison of Models

Table 3 summarizes the predictive performance of various machine learning models for energy-response prediction in the sheet metal forming problem. The comparison includes conventional regression-based models, neural-network-based approaches, the proposed physics-consistent Energy-Informed Neural Network (EINN), and an additional ensemble-learning-based model. The ensemble model was included because ensemble learning has been shown to improve predictive robustness and accuracy in nonlinear engineering problems by leveraging the complementary strengths of multiple learners [32]. To provide a stronger baseline, a voting ensemble regressor was incorporated. The model combines multiple tree-based regressors, including Random Forest, Extra Trees, XGBoost, and LightGBM, and generates the final prediction by averaging their outputs. This approach leverages complementary learning characteristics of different models to improve predictive robustness.

All reported values represent the mean ± standard deviation over ten independent runs (2-fold cross-validation with five random seeds per fold). Among the conventional machine learning models, Ridge regression, ExtraTrees, ElasticNet, and RandomForest exhibit moderate and relatively stable performance, achieving RMSE values in the range of approximately 0.018–0.021 and R^2^ values between 0.76 and 0.87. These results indicate that linear and ensemble-based methods are capable of capturing global energy-response trends to a certain extent, even under limited simulation data. In contrast, SVR and KNN show noticeably degraded performance, with RMSE values exceeding 0.03 and R^2^ around 0.58. HistGBDT (MultiOutput) fails to generalize under small-data conditions, yielding a negative mean R^2^ (−0.220 ± 0.357), suggesting instability and structural mismatch for coupled multi-target energy prediction. A baseline unconstrained MLP with the same architecture as the proposed EINN was evaluated for comparison. It showed lower predictive performance than both the proposed EINN and the energy-consistency–only model. To isolate the effect of physics-based constraints, an MLP augmented only with energy-consistency constraints (without the Fenchel–Young term) was additionally evaluated. This model achieves RMSE = 0.0192 ± 0.0098 and R^2^ = 0.6515 ± 0.6693. Although the mean error improves compared to the baseline MLP, the large standard deviation indicates unstable convergence and inconsistent generalization across runs. This suggests that simple constraint regularization alone is insufficient to structurally stabilize the learning process. In contrast, the proposed EINN incorporating both energy constraints and the Fenchel–Young-based duality loss demonstrates a substantial and statistically stable performance improvement. The EINN achieves: RMSE = 0.0096 ± 0.0021, MAE = 0.0065 ± 0.0014, R^2^ = 0.9778 ± 0.0096. Compared to the best-performing conventional ML model (Ridge, RMSE ≈ 0.0185), the proposed EINN reduces prediction error by approximately 48% while significantly improving explanatory power. Moreover, the standard deviation of R^2^ (±0.0096) remains small relative to other neural-network configurations, indicating consistent convergence behavior across repeated runs. Importantly, the architectural capacity of the EINN is identical to that of the baseline MLP. Therefore, the observed performance gain cannot be attributed to increased model complexity. Instead, the improvement arises from the enforcement of dual physical structure through the Fenchel–Young loss term, which restricts the admissible solution space to physically consistent configurations. This structural regularization reduces optimization instability and suppresses physically inconsistent solutions that may otherwise emerge in purely data-driven regression. The repeated 10 runs evaluation further confirms the statistical robustness of the proposed approach. The relatively low variance in RMSE and R^2^ demonstrates that the performance gain is not an artifact of favorable initialization or data splitting. Rather, embedding energy-consistency constraints together with the Fenchel–Young-based dual formulation stabilizes the empirical risk landscape, guiding optimization toward physically admissible regions and enhancing generalization reliability under small-data conditions. Taken together, these results suggest that conventional regression models are limited in their ability to capture the coupled and path-dependent nature of energy evolution in sheet metal forming. In contrast, the proposed EINN effectively embeds the underlying physical structure into the learning process, yielding both improved accuracy and statistically stable performance.

### 3.4. Effect of the Fenchel–Young Loss Term on Learning Stability and Physical Consistency

To examine how the Fenchel–Young loss term modifies learning behavior and whether it effectively reduces physically inconsistent predictions, we analyze per-variation-variable performance and variance across repeated runs. The proposed EINN (energy-consistency loss + Fenchel–Young loss) consistently outperforms baseline models across all variation variables. The improvement is particularly pronounced for global energy variables directly involved in structural coupling.

For E_total_end (total system energy at the final forming step), conventional models show RMSE values around 0.028–0.031 (Ridge: 0.0286; ElasticNet: 0.0280; RandomForest: 0.0311), while the energy-consistency-only MLP achieves 0.0236. In contrast, the proposed EINN reduces the RMSE to 0.0138, corresponding to approximately a 50% error reduction relative to strong linear baselines. A similar pattern is observed for max_E_total (maximum total energy during forming), where the RMSE decreases from approximately 0.0286 (Ridge) to 0.0139 under the proposed formulation. Beyond mean accuracy, variance across repeated runs is substantially reduced. For E_total_end, the R^2^ standard deviation decreases from 0.043 (energy-consistency-only MLP) to 0.006 in the proposed EINN. This indicates that the Fenchel–Young loss suppresses unstable solution branches and guides optimization toward structurally admissible regions of the hypothesis space. The largest improvements are observed for variation variables strongly coupled through physical duality relations. For IE_end (internal energy at the final step), the RMSE decreases from 0.0121 (Ridge) and 0.0131 (ExtraTrees) to 0.00589 under the proposed model, representing more than a 50% reduction in prediction error. For dynamically coupled variables such as Vz_end (final punch velocity in the forming direction), conventional models exhibit limited predictive capability due to the strong coupling between energy and kinematic variables (ElasticNet R^2^ = 0.14; SVR R^2^ = −0.20; HistGBDT R^2^ < 0; energy-consistency-only MLP R^2^ = −0.038). In contrast, the proposed EINN achieves consistently high predictive accuracy, with R^2^ = 0.970 ± 0.018. A similar trend is observed for max_KE (maximum kinetic energy during forming), where the RMSE is substantially reduced compared with conventional baseline models, indicating improved learning of energy–momentum coupling. Baseline models occasionally produce physically inconsistent predictions, which may appear as negative R^2^ values or large variance across repeated runs. The unconstrained baseline MLP shows degraded performance (R^2^ = 0.2299 ± 0.5399), indicating relatively large performance variability across repeated runs under small-data conditions. The energy-consistency–only MLP improves the mean predictive accuracy but still exhibits relatively high variance (R^2^ std = 0.6693), suggesting that constraint regularization alone does not fully stabilize optimization across repeated runs. In contrast, the proposed EINN maintains consistently high predictive performance across all evaluated variables, with R^2^ standard deviation below 0.01 in the overall evaluation and no extreme outliers across repeated runs. These results indicate that the Fenchel–Young loss helps stabilize the learning process by enforcing physically consistent coupling relationships during training. The Fenchel–Young loss embeds the dual structure between energy, momentum, and velocity directly into the optimization objective. Rather than minimizing residual errors independently for each output, the network is constrained to satisfy structural coupling during training. Consequently, the empirical risk landscape becomes structurally regularized, leading to reduced variance, improved prediction of dynamically coupled variables, and elimination of unstable physically inadmissible solutions. The improvement therefore arises not merely from error minimization, but from restricting the hypothesis space to physically consistent configurations.

## 4. Conclusions

Overall, this study establishes an energy-consistent learning framework for accurate energy-response prediction in sheet metal forming under small-data conditions. Quantitatively, the proposed EINN (Constraint + Fenchel–Young) achieves a substantial improvement over the best-performing conventional baseline. Compared with Ridge regression (the strongest non-physics baseline in terms of RMSE), EINN reduces RMSE from 0.0185 to 0.0096 (approximately 48% improvement) and MAE from 0.0153 to 0.0065 (approximately 57% improvement), while increasing the mean R^2^ from 0.874 to 0.978. Compared with an energy-constrained MLP without the Fenchel–Young term, the RMSE is reduced by approximately 50%, demonstrating the critical contribution of the additional physics-consistent regularization.

From an industrial perspective, these improvements are significant because energy-response prediction can serve as a fast and stable surrogate model to replace repeated high-cost finite element simulations during early-stage process exploration. In practical forming design, the proposed framework can support rapid screening of process parameters, reduction in computational resource requirements, and more reliable identification of physically meaningful forming conditions. Unlike conventional PINN approaches that may require dense spatiotemporal supervision or suffer from instability under limited data, the proposed energy-consistent structure provides a lightweight and robust alternative tailored to small-data industrial scenarios. With increased data availability, the framework may further scale to more complex forming configurations and multi-physics extensions. Nevertheless, several limitations remain. First, the present validation is primarily simulation-based, and robustness under real measurement noise has not yet been fully verified. Second, the current framework focuses on global energy-related quantities and does not explicitly model local field variables such as stress localization or damage evolution. Third, the dataset size and diversity may limit generalization across broader material and friction conditions. Future work will therefore focus on systematic experimental validation, extension to more complex forming processes, and integration of additional physically meaningful quantities to further enhance industrial applicability and interpretability. From an industrial perspective, the proposed framework can be used as a surrogate model to replace repeated FEM simulations during early-stage process exploration. Engineers can utilize the predicted energy responses to evaluate forming conditions, assess process stability, and identify physically feasible parameter ranges. The predicted variables are physically interpretable, including energy components and kinematic quantities, which are directly related to forming quality and deformation behavior. Furthermore, the Fenchel–Young constraint ensures physically consistent predictions, enhancing the reliability of the model in practical applications. However, the present study is limited to simulation-based data and does not explicitly account for measurement noise or broader material variability, which should be addressed in future work.

## Figures and Tables

**Table 1:** Conceptual comparison between classical PINNs, energy-based learning frameworks, previously proposed EINN approaches, and the proposed Fenchel–Young-based EINN.

Framework	Physics Enforcement Mechanism	Derivative Requirement	Primary Objective	Structural/Theoretical Basis	Representative References
Classical PINNs	Strong-form PDE residuals embedded in loss	High-order automatic differentiation	Forward solution approximation	Governing equations explicitly enforced	Raissi et al. (2019) [23]
General Energy-Based Learning	Implicit energy function parameterization	None (data-driven)	Energy score minimization	Often heuristic, no explicit convex structure	LeCun et al. (2006) [24]
Previous EINN Approaches	Gradient-based regularization guided by expert knowledge	First-order gradients	Robust regression under small data	Empirically motivated constraint design	Quagliato et al. (2025) [16]
Proposed EINN (This Work)	Fenchel–Young dual energy consistency constraint	No PDE residual; dual-variable compatibility enforcement	Energy-consistent prediction and process-variable exploration	Convex-duality-based formulation	-

**Table 2:** Mechanical properties of metallic materials used in finite element simulations for dome forming analysis (source: Matweb.org).

Material	Density ρ (g/cm^3^)	Young’s Modulus E (GPa)	Yield Strength (MPa)
Al 1100	2.70	70	35
STS304	7.93	193	205
GI	7.85	210	270
SPCC	7.85	205	280
SS400	7.85	200	250
AZ31B	1.77	45	190
Cu	8.96	110	70
Brass	8.40	97	200
SPTE	7.85	210	280
Ti Grade 2	4.51	105	275
DP590	7.85	210	590
BeCu	8.25	128	450
Inconel 718	8.19	200	1035
Mild Steel (SS41)	7.85	210	240
AA5052-O	2.68	70.3	89.6
AA6061-T4	2.70	69	145
AA7075-T6	2.81	71.7	503
DC01	7.85	210	140

**Table 3:** Performance comparison of baseline ML models and the proposed EINN variants for energy-response prediction (mean ± std over 10 runs).

Model	RMSE (Mean ± Std)	MAE (Mean ± Std)	R^2^ (Mean ± Std)
EINN (Constraint + Fenchel–Young)	0.0096 ± 0.0021	0.0065 ± 0.0014	0.9778 ± 0.0096
MLP (Energy constraints without Fenchel–Young)	0.0192 ± 0.0098	0.0136 ± 0.0055	0.6515 ± 0.6693
Ridge	0.0185 ± 0.0022	0.0153 ± 0.0019	0.8745 ± 0.0301
ElasticNet	0.0185 ± 0.0025	0.0151 ± 0.0021	0.7655 ± 0.0353
ExtraTrees	0.0181 ± 0.0065	0.0128 ± 0.0039	0.8555 ± 0.0576
VotingEnsemble	0.0183 ± 0.0071	0.0136 ± 0.0049	0.8748 ± 0.0479
RandomForest	0.0209 ± 0.0068	0.0157 ± 0.0046	0.8424 ± 0.0659
SVR (RBF, MultiOutput)	0.0329 ± 0.0153	0.0246 ± 0.0116	0.5786 ± 0.1758
KNN	0.0448 ± 0.0118	0.0355 ± 0.0103	0.5842 ± 0.1474
HistGBDT (MultiOutput)	0.0851 ± 0.0102	0.0757 ± 0.0078	−0.2205 ± 0.3575
MLP (baseline)	0.0495 ± 0.0283	0.0419 ± 0.0254	0.2299 ± 0.5399

## Data Availability

The original contributions presented in this study are included in the article. Further inquiries can be directed to the corresponding author.

## References

[1] Marques A.E., Parreira T.G., Pereira A.F., Ribeiro B.M., Prates P.A. (2024). Machine learning applications in sheet metal constitutive modelling: A review. Int. J. Solids Struct..

[2] Zhang R., Shao Z., Lin J. (2018). A review on modelling techniques for formability prediction of sheet metal forming. Int. J. Lightweight Mater. Manuf..

[3] Marques A.E., Dib M.A., Khalfallah A., Soares M.S., Oliveira M.C., Fernandes J.V., Ribeiro B.M., Prates P.A. (2022). Machine learning for predicting fracture strain in sheet metal forming. Metals.

[4] Tarraf A., Manthey K., Mohammadi S.A., Martin P., Moj L., Burak S., Park E., Terboven C., Wolf F. (2025). When AI bends metal: AI-assisted optimization of design parameters in sheet metal forming. arXiv.

[5] Munzone F., Hazrati J., Hakvoort W., van den Boogaard T. Comparative study of artificial neural network and physics-informed neural network application in sheet metal forming. Proceedings of the 27th International ESAFORM Conference on Material Forming (ESAFORM 2024).

[6] Thamm A., Thamm F., Sawodny A., Zeitler S., Merklein M., Maier A. (2023). Unsupervised deep learning for advanced forming limit analysis in sheet metal: A tensile test-based approach. Materials.

[7] Lee S., Hyun D., Hong S. AI-based prediction of cracks and wrinkles in sheet metal forming due to drawbeads. Proceedings of the Korean Society for Technology of Plasticity Conference, Jeju, Republic of Korea.

[8] Choi K.K., Kim N. (2001). Prediction of forming limit diagram dependent on strain history in sheet metal forming. Trans. Korean Soc. Mech. Eng. A.

[9] Arrieux R. (1997). Determination and use of the forming limit stress surface of orthotropic sheets. J. Mater. Process. Technol..

[10] Safari M., Hosseinipour S.J., Azodi H.D. (2011). Experimental and numerical analysis of forming limit diagram (FLD) and forming limit stress diagram (FLSD). Mater. Sci. Appl..

[11] Mesmer G., Colton J. (2024). Variation of the friction conditions in cold ring compression tests of aluminum 1100-O and aluminum 6061-T6 as applied to room temperature forging. Cogent Eng..

[12] Rao K.P., Wei J.J. (2001). Performance of a new dry lubricant in the forming of aluminum alloy sheets. Wear.

[13] Ju L., Mao T., Malpica J., Altan T. (2015). Evaluation of lubricants for stamping of Al 5182-O aluminum sheet using cup drawing test. J. Manuf. Sci. Eng..

[14] Trzepieciński T., Slota J., Kaščák Ľ., Gajdoš I., Vojtko M. (2023). Friction behaviour of 6082-T6 aluminium alloy sheets in a strip draw tribological test. Materials.

[15] Sabet A.S., Domitner J., Ristić A., Öksüz K.I., Ripoll M.R., Sommitsch C. (2023). Effects of temperature on friction and degradation of dry film lubricants during sliding against aluminum alloy sheets. Tribol. Int..

[16] Quagliato L., Perin M., Modanloo V., Lee T. Expert-informed neural network (EINN) for the forming depth prediction from a small-scale sheet metal forming database. Proceedings of the 28th International ESAFORM Conference on Material Forming, ESAFORM 2025.

[17] Bai J., Lin Z., Wang Y., Wen J., Liu Y., Rabczuk T., Gu Y.T., Feng X.Q. (2025). Energy-based physics-informed neural network for frictionless contact problems under large deformation. Comput. Methods Appl. Mech. Eng..

[18] El Mrabti I., El Hakimi A., Touache A., Chamat A. (2022). A comparative study of surrogate models for predicting process failures during the sheet metal forming process of advanced high-strength steel. Int. J. Adv. Manuf. Technol..

[19] Guo M., Haghighat E. (2022). Energy-based error bound of physics-informed neural network solutions in elasticity. J. Eng. Mech..

[20] Liu S., Xia Y., Shi Z., Yu H., Li Z., Lin J. (2021). Deep learning in sheet metal bending with a novel theory-guided deep neural network. IEEE/CAA J. Autom. Sin..

[21] Wang Y., Li F., Li Y., Li J., Chen H., Bai Y. (2025). Effects of ultrasonic surface rolling process on microstructure and mechanical properties of metastable Fe50Mn30Co10Cr10 high-entropy alloy fabricated by laser directed energy deposition. J. Mater. Res. Technol..

[22] Vaiyapuri T., Elashmawi W.H., Asiedu W. (2025). VAE-CNN: Deep Learning on Small Sample Dataset Improves Hydrogen Yield Prediction in Co-gasification. J. Comput. Cogn. Eng..

[23] Raissi M., Perdikaris P., Karniadakis G.E. (2019). Physics-informed neural networks: A deep learning framework for solving forward and inverse problems involving nonlinear partial differential equations. J. Comput. Phys..

[24] LeCun Y., Chopra S., Hadsell R., Ranzato M., Huang F. (2006). A tutorial on energy-based learning. Predict. Struct. Data.

[25] Davey S., Das R., Cantwell W.J., Kalyanasundaram S. (2013). Forming studies of carbon fibre composite sheets in dome forming processes. Compos. Struct..

[26] Guo M., Han W., Zhong H. (2016). Legendre–Fenchel duality and a generalized constitutive relation error. arXiv.

[27] Blondel M., Martins A., Niculae V. Learning classifiers with Fenchel–Young losses: Generalized entropies, margins, and algorithms. Proceedings of the 22nd International Conference on Artificial Intelligence and Statistics (AISTATS 2019).

[28] Blondel M., Llinares-López F., Dadashi R., Hussenot L., Geist M. Learning energy networks with generalized Fenchel–Young losses. Proceedings of the 36th International Conference on Neural Information Processing Systems.

[29] Qi B., Gong W., Li L. (2025). Understanding deep learning optimization through Fenchel–Young loss: Theory and evidence. SSRN.

[30] Nielsen F. (2022). Statistical divergences between densities of truncated exponential families with nested supports: Duo Bregman and duo Jensen divergences. Entropy.

[31] Chodrow P.S. (2025). Equivalence of Informations Characterizes Bregman Divergences. Entropy.

[32] Asgarkhani N., Kazemi F., Jankowski R., Formisano A. (2025). Dynamic ensemble-learning model for seismic risk assessment of masonry infilled steel structures incorporating soil-foundation-structure interaction. Reliab. Eng. Syst. Saf..

